# Detection of novel orthoparamyxoviruses, orthonairoviruses and an orthohepevirus in European white-toothed shrews

**DOI:** 10.1099/mgen.0.001275

**Published:** 2024-08-01

**Authors:** Viola C. Haring, Benedikt Litz, Jens Jacob, Michael Brecht, Markus Bauswein, Julia Sehl-Ewert, Marta Heroldova, Claudia Wylezich, Donata Hoffmann, Rainer G. Ulrich, Martin Beer, Florian Pfaff

**Affiliations:** 1 Friedrich-Loeffler-Institut, Institute of Novel and Emerging Infectious Diseases, Greifswald - Insel Riems, Germany; 2 Friedrich-Loeffler-Institut, Institute of Diagnostic Virology, Greifswald - Insel Riems, Germany; 3 Julius Kühn-Institute, Institute for Epidemiology and Pathogen Diagnostics, Rodent Research, Muenster, Germany; 4 Bernstein Center for Computational Neuroscience Berlin, Humboldt-Universität zu Berlin, Berlin, Germany; 5 Institute of Clinical Microbiology and Hygiene, Regensburg University Hospital, Regensburg, Germany; 6 Friedrich-Loeffler-Institut, Department of Experimental Animal Facilities and Biorisk Management, Greifswald - Insel Riems, Germany; 7 Department of Forest Ecology, Faculty of Forestry and Wood Technology, Mendel University in Brno, Brno, Czech Republic

**Keywords:** *Crocidura*, insectivores, metagenome, reservoir, virome, white-toothed shrews

## Abstract

While the viromes and immune systems of bats and rodents have been extensively studied, comprehensive data are lacking for insectivores (order Eulipotyphla) despite their wide geographic distribution. Anthropogenic land use and outdoor recreational activities, as well as changes in the range of shrews, may lead to an expansion of the human–shrew interface with the risk of spillover infections, as reported for Borna disease virus 1. We investigated the virome of 45 individuals of 4 white-toothed shrew species present in Europe, using metagenomic RNA sequencing of tissue and intestine pools. Moderate to high abundances of sequences related to the families *Paramyxoviridae*, *Nairoviridae*, *Hepeviridae* and *Bornaviridae* were detected. Whole genomes were determined for novel orthoparamyxoviruses (*n*=3), orthonairoviruses (*n*=2) and an orthohepevirus. The novel paramyxovirus, tentatively named Hasua virus, was phylogenetically related to the zoonotic Langya virus and Mòjiāng virus. The novel orthonairoviruses, along with the potentially zoonotic Erve virus, fall within the shrew-borne Thiafora virus genogroup. The highest viral RNA loads of orthoparamyxoviruses were detected in the kidneys, in well-perfused organs for orthonairoviruses and in the liver and intestine for orthohepevirus, indicating potential transmission routes. Notably, several shrews were found to be coinfected with viruses from different families. Our study highlights the virus diversity present in shrews, not only in biodiversity-rich regions but also in areas influenced by human activity. This study warrants further research to characterize and assess the clinical implications and risk of these viruses and the importance of shrews as reservoirs in European ecosystems.

**Impact Statement**

The detection of the zoonotic Langya virus in white-toothed shrews in China, as well as studies on the zoonotic Borna disease virus 1 in bicolored white-toothed shrews in Germany, have stimulated interest in white-toothed shrews as reservoirs for pathogens. Here, we used metagenomic sequencing to reveal the virome of white-toothed shrews in Europe. This has resulted in the description and phylogenetic classification of several novel viruses of the families *Paramyxoviridae*, *Nairoviridae* and *Hepeviridae*. We could demonstrate a high diversity of viruses and co-infections in synanthropic white-toothed shrews. Public awareness of pathogens in shrews is important for establishing targeted countermeasures for risk reduction while maintaining biodiversity.

## Data Summary

Viral genomes and raw read data were uploaded to GenBank using the accessions OR713845–OR713892 (BioProject: PRJNA1028379).

## Introduction

**Fig. 1.**
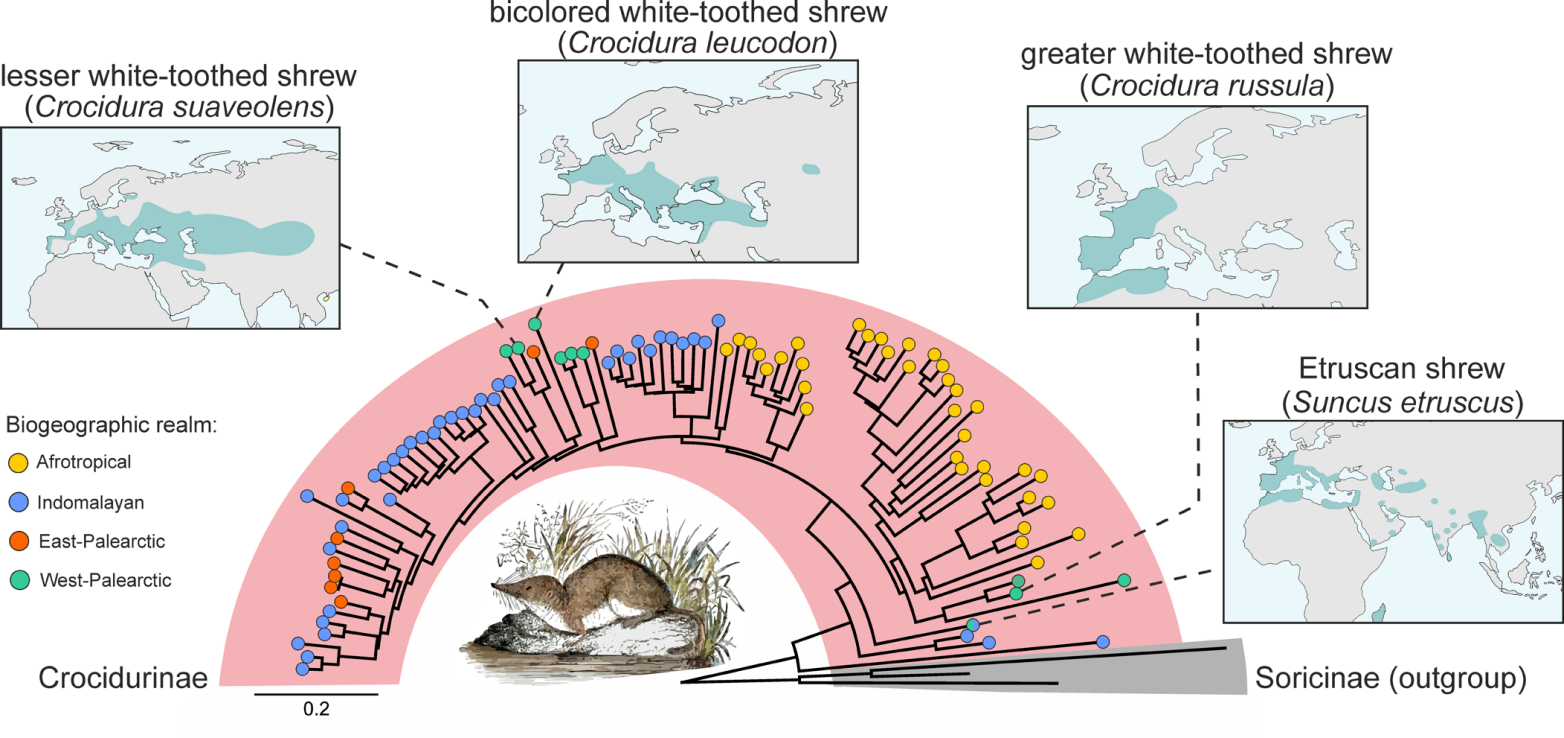
Phylogenetic relationships and biogeographic distribution of extant white-toothed shrews. The phylogenetic tree is based on all available *cytochrome b* sequences from white-toothed shrews (subfamily Crocidurinae) and a selected outgroup of red-toothed shrews (subfamily Soricinae) (IQ-TREE2; version 2.2.2.6). The biogeographic distribution of these animals can be broadly grouped into four realms: Afrotropical, Indomalayan, East-Palearctic and West-Palearctic. The geographical range of the four Crocidurinae species that can be found in Europe is highlighted in the maps, according to [8]. Note the phylogenetic distances between *Suncus etruscus*, *Crocidura russula* and *Crocidura leucodon*/*Crocidura suaveolens*.

Knowledge of pathogen diversity in wildlife species is essential to be prepared for the next pandemic, a key task of modern virology [1]. Current estimates suggest that 75% of emerging human pathogens originate from (wild) animals [2,3]. Small mammals, especially rodents and bats, are well-known reservoirs of zoonotic viruses [4,6], but little is known about the virosphere of insectivore species, especially shrews [7]. Shrews (Mammalia: Eulipotyphla: Soricidae) are species-rich and phylogenetically ancient (>45 million years) [8]. Three subfamilies are defined within the family Soricidae: Soricinae (red-toothed shrews), Crocidurinae (white-toothed shrews) and Myosoricinae (African white-toothed shrews). At least 242 species from 10 genera with an almost global distribution belong to the Crocidurinae subfamily, and the great diversity is increasing with the discovery of new species (Fig. 1) [9].

Primarily, four synanthropic species of white-toothed shrews are found in Europe: the bicolored white-toothed shrew (*Crocidura leucodon*), the greater white-toothed shrew (*Crocidura russula*), the lesser white-toothed shrew (*Crocidura suaveolens*) and the Etruscan shrew (*Suncus etruscus*) [8]. *C. russula* originates from North Africa and is currently distributed across Western Europe towards Fennoscandia and the Czech Republic [10,12]. *C. leucodon* is found from northern France through southern Europe to the Caspian Sea. The Etruscan shrew, one of the smallest recent living mammals with a body weight <2 g, is found mainly in southern Europe with a scattered distribution across parts of Africa and Asia (Fig. 1) [8]. The phylogenetic relationships among shrew species remain incompletely understood, with several species complexes, including the *C. suaveolens* sf. species complex, which shows a wide but fragmented distribution from the Atlantic coast to China [8].

At present, knowledge of pathogens in European shrews, especially white-toothed shrews, is limited, apart from intensive studies of *C. leucodon*, the natural reservoir for zoonotic Borna disease virus 1 (BoDV-1) [13], which causes fatal encephalitis in both humans and domestic animals [14,15]. However, sporadic detection of orthonairoviruses and paramyxoviruses has been reported.

Since the first report of Thiafora virus (TFAV) isolated from a *Crocidura* sp. shrew in Senegal in 1971, the number of new orthonairoviruses detected in shrews increased [16]. Erve virus (ERVEV) was identified in *C. russula* from France [16,18]. More recently, Lamusara virus and Lamgora virus have been described in the Goliath shrew (*Crocidura goliath*) from Gabon [19] and Cencurut virus (CENV) in the Asian house shrew (*Suncus murinus*) from Singapore [20]. All these viruses belong to the Thiafora virus genogroup, which is distantly related to the zoonotic Crimean-Congo haemorrhagic fever virus (CCHFV). CCHFV causes highly contagious haemorrhagic fever in humans, with a case fatality rate up to 40% [21]. It is transmitted by ticks (*Hyalomma* spp.) or by direct contact to viraemic humans and animals. A small mammal reservoir for CCHFV has been discussed, but not identified. Ticks are now considered both reservoirs and amplifying hosts [21].

Recently, the zoonotic Langya virus (LayV, family *Paramyxoviridae*) was isolated from febrile human patients and detected in Ussuri white-toothed shrews (*Crocidura lasiura*) and Shantung white-toothed shrews (*Crocidura shantungensis*) in China [22]. Gamak virus (GamV) and Daeryong virus have been identified in *C. lasiura* and *C. shantungensis* in Asia, respectively [23]. Recent studies in Belgium identified Melian virus (MeliV) in African large-headed shrews (*Crocidura grandiceps*), Denwin virus (DewV) in European *C. russula* and Ninorex virus in the Eurasian pygmy shrew (*Sorex minutus*), a red-toothed shrew species [24,25]. Classification of the shrew-associated paramyxoviruses as a separate genus *Parahenipavirus* was proposed and accepted by the International Committee on Taxonomy of Viruses (ICTV) [26]. Parahenipaviruses are phylogenetically related to the members of the genus *Henipavirus*, which includes the highly contagious and lethal zoonotic Hendra virus (HeV) and Nipah virus (NiV) discovered in fruit bats in Australia and Southeast Asia, respectively [27,28].

Here, we investigated the virome of four white-toothed shrew species present in Europe (total *n*=45) using a straightforward sample-pooling approach, followed by high-throughput RNA sequencing and specific reverse transcription quantitative PCR (RT-qPCR) confirmation and determination of the viral tissue distribution to indicate potential transmission routes. Our study is thus one of the first to record specifically on the virome of white-toothed shrews in Europe. The surprisingly high number of novel viruses suggests a previously underestimated reservoir function of shrews, which might be even greater than that of sympatric rodent and bat species as recently postulated [29], and this is not only in subtropical but also in temperate regions.

## Methods

### Sample selection and RNA extraction

A total of 19 bicolored white-toothed shrews (*C. leucodon*), 16 greater white-toothed shrews (*C. russula*), and 6 lesser white-toothed shrews (*C. suaveolens*) covering the known distribution of these species in Germany, captured between 2002 and 2021, and two additional *C. leucodon* collected in the Czech Republic in 2007 were selected. In addition, two Etruscan shrews (*S. etruscus*) from a German breeding colony were included (Fig. S1 and Table S1, available in the online Supplementary Material 1 and Supplementary Material 2). Identification of the shrew species was based on molecular analysis of the *cytochrome b* gene as described previously [30].

First, organ tissues were pooled per individual, consisting of small pieces of brain, lung, spleen, liver and kidney tissues, as available. These tissue pools were directly immersed in 1 ml QIAzol (Qiagen, Germany) and stored at −80 °C until further processing. In addition, intestine tissue samples containing ingesta from several individuals of the same species were pooled and processed according to the individual tissue pools (Table S1).

Tissue pools were homogenized for 2 min at 30 Hz using 5 mm steel beads on a TissueLyser II instrument (Qiagen, Germany). Chloroform (Carl Roth, Germany) was added to each reaction, mixed vigorously and centrifuged at 13,000×g for 10 min. The upper aqueous phase was further processed for total RNA extraction using the Agencourt RNAdvance Tissue Kit (Beckman Coulter, Germany) on a KingFisher Flex Purification System (Thermo Fisher Scientific, Germany) according to the manufacturer’s instructions.

### Library preparation from RNA and high-throughput sequencing

Total RNA quantity was measured using a NanoDrop ND1000 UV spectrophotometer (Peqlab, Germany), and total RNA quality was assessed using a 4150 TapeStation system (Agilent, Germany). In an attempt to reduce the amount of host-derived ribosomal RNA (rRNA), total RNA was treated with the ‘pan mammalia’ riboPOOL ribosomal depletion kit (siTOOLs Biotech, Germany) according to the manufacturer’s instructions. The rRNA-depleted total RNA was then used for library preparation using the Collibri Stranded RNA Library Prep Kit for Illumina Systems (Invitrogen, Germany) according to the manufacturer’s instructions. Final libraries were quantified using a Qubit 2.0 fluorometer in conjunction with the Qubit dsDNA HS Assay-Kit (Invitrogen, Germany). The libraries were then pooled, submitted to CeGaT GmbH (Germany) and sequenced on a NovaSeq 6000 system (Illumina, USA) in 1×100 base pair (bp) mode.

### Capture enrichment of high-throughput sequencing libraries

The high-throughput sequencing libraries were further enriched for specific sequences of epizootic and zoonotic viruses using biotinylated RNA baits (VirBaits panel) [31]. In the present study, we used the extended VirBaits 2.0, which contains oligonucleotide baits for the viruses as described by Wylezich *et al.* [31] supplemented with baits for hepatovirus A, hepatitis B virus, hepacivirus C, orthohepevirus A, infectious pancreatic necrosis virus, Zika virus, Usutu virus, Japanese encephalitis virus, yellow fever virus, Kyasanur forest disease virus, tick-borne encephalitis virus, dengue virus, enterovirus C, measles virus, mumps virus, salmonid novirhabdovirus, viral haemorrhagic septicemia virus, Marburg virus, Sindbis virus, chikungunya virus, rubella virus, rustrela virus, La Crosse virus, Lassa mammarenavirus, orthohantaviruses and betacoronaviruses (VirBaits 2.0 one health panel). The VirBaits 2.0 collection comprises 134,710 RNA baits, each with a length of 80 nucleotides. The VirBaits 2.0 panel was implemented following the guidelines provided by the manufacturer (standard protocol of the myBaits manual v.5.00, Arbor Biosciences, September 2020) for 24 h at 60 °C.

The enriched libraries were finally amplified using the Collibri Library Amplification Master Mix (Invitrogen, Germany, 14 cycles) and sequenced on an iSeq system (Illumina, USA) in 2×150 bp mode.

### Sequence data analysis

Raw reads were first trimmed for adapter contamination and poor quality using Trim Galore (version 0.6.10) in automatic adapter detection mode. Subsequently, host-specific background was then removed from the trimmed libraries using BBMap (version 39.01, *k*=13 [32]) together with the combined genomic assemblies of *Crocidura indochinensis* (Indochinese white-toothed shrew, GCA_004027635.1), *S. etruscus* (GCF_024139225.1), *Sorex fumeus* (smokey shrew, GCA_026122425.1), *Sorex araneus* (common shrew, GCF_000181275.1) and *Cryptotis parvus* (North American least shrew, GCA_021461705.1) as reference. In addition, rRNA-derived reads were removed using SortMeRNA (version 4.3.6 [33]) with all rRNA entries of the SILVA database (release 138.1 [34]) belonging to the taxon ‘Vertebrata’ as reference.

The trimmed and host sequence-depleted libraries were individually assembled *de novo* using rnaSPAdes (version 3.15.5 [35]). The metatranscriptomic pipeline SqueezeMeta (version 1.6.2 [36]) was also used for *de novo* assembly, taxonomic classification and quantification. Specifically, SqueezeMeta was run with the option ‘-contiglen 400’ in ‘seqmerge’ mode, which merges individual assemblies into a single combined assembly prior to further processing. The assembly was then trimmed with regard to poly(A) and poly(T) sequences at the end or start of the contigs, using cutadapt (version 4.0 [37]). This step prevents unspecific mapping to poly(A)-tails. The trimmed *de novo* assembled contigs were then used for a final run of SqueezeMeta using the ‘-extassembly’ option.

### Selection of complete viral genomes

Contigs that were classified as viral sequences and likely represented full genomes were selected based on their size from the SqueezeMeta assembly and compared with the rnaSPAdes assembly. For the final quality check, the raw reads were mapped to the likely full genomes using the Geneious Prime (version 2021.0.1) generic mapper. Open reading frame (ORF) annotation was done in Geneious Prime using appropriate references and the ‘Find ORFs’ function. For selected samples, we re-sequenced tissue pool-derived libraries and analysed them together with sequences obtained from individual kidney samples in order to improve the coverage of the identified genomes.

### Sequencing of viral RNA 5′ends

SuperScript III reverse transcriptase (Invitrogen, Germany) and virus-specific primers were used to generate cDNA from the 5′ end of selected virus genomes. The cDNA was then further amplified using the 5′ Rapid Amplification of cDNA Ends (RACE) 2.0 system (Invitrogen, Germany). Final PCR products were prepared for sequencing using BigDye Terminator v1.1 (Applied Biosystems, USA) and sequenced on a 3500 Genetic Analyzer (Applied Biosystems, USA).

### Phylogenetic analysis of complete viral genomes

Viral sequences were aligned with publicly available reference sequences using MUSCLE (version 3.8.425). Maximum-likelihood phylogenetic trees were calculated using IQ-TREE2 (version 2.2.2.6 [38]) with an automated model selection and each 100 000 ultra-fast bootstrap [39] and SH-aLRT [40] replicates.

In detail, for hepevirus phylogeny, we selected 36 representative genomes of the subfamily *Orthohepevirinae* and five genomes of fish hepeviruses (subfamily *Parahepevirinae*) as references for phylogenetic analysis. The first 450 amino acid (aa) residues of the ORF1-encoded non-structural polyprotein wasere aligned and used for phylogeny.

For paramyxovirus phylogeny, we selected 54 representative genomes of the subfamily *Orthoparamyxovirinae* and one genome of the subfamily *Metaparamyxovirinae* as references for phylogenetic analysis. The aa sequences of the large protein (L, including RNA-directed RNA polymerase, capping and cap methylation activities) were aligned and used for phylogeny.

For nairovirus phylogeny, we selected 46 representative genomes of the genus *Orthonairovirus* and one genome of the genus *Shaspivirus* as references for phylogenetic analysis. The aa sequences of the large protein (L, large segment, containing an RNA-directed RNA polymerase domain) were aligned and used for phylogeny.

For bornavirus phylogeny, we selected 74 shrew and domestic animal-derived genomes of BoDV-1. Borna disease virus 2 (BoDV-2) was used as the outgroup. Nucleotide sequences spanning the nucleoprotein (N), phospho- (P) and X protein-coding sequences were aligned and used for phylogenetic analysis.

### Virus-specific RT-qPCR

Primers and probes for RT-qPCR detection of viral RNA of the detected nairo-, paramyxo- and hepeviruses were designed using Primer3web (version 4.1.0 [41]). The L ORF was targeted for nairoviruses and paramyxoviruses and ORF3 for hepeviruses. For specific detection of BoDV-1, the BoDV-1-Mix1-FAM assay was used [42]. A set of primers and probes targeting the ß-actin-2 gene was used as an internal control [43]. Primer and probe sequences are shown in Table S3. The RT-qPCRs were performed using the AgPath-ID One-Step RT-PCR Kit (Applied Biosystems, USA) according to the manufacturer’s instructions and run on a CFX96 Touch Real-Time PCR Detection System (Bio-Rad, Germany) with the following protocol: 10 min at 45 °C for reverse transcription, 10 min at 95 °C for polymerase activation, 42 cycles of 15 s at 95 °C, 20 s at 57 °C (with fluorescence detection during this step) and 30 s at 72 °C.

### Tissue distribution of novel viruses

An organ tissue panel was prepared from selected animals to assess the tissue distribution of viral RNA. Approximately 50 mg of tissue was homogenized in 500 µl phosphate-buffered saline (PBS) for 2 min at 30 Hz using 5 mm steel beads on a TissueLyser II instrument (Qiagen, Germany). Total nucleic acids were extracted using the NucleoMag Vet Kit (Macherey and Nagel, Germany) on a KingFisher Flex Purification System (Thermo Fisher Scientific, Germany) according to the manufacturer’s instructions.

### Virus isolation in cell culture

For cell culture isolation of Rasenna virus from *S. etruscus*, organ tissue material was lysed in a cell culture medium and used to inoculate Vero cells (CCLV-RIE 0228) or baby hamster kidney (BHK) 21 cells (CCLV-RIE 0179) in a TC12.5 format (serum-free cell culture medium plus antibiotics). The cell culture supernatant from each cell culture flask was used for passaging to achieve four consecutive passages. In addition, the cells were passaged again separately to obtain four consecutive passages. Tissues used for the different isolation attempts included the liver, spleen, heart, muscle, fat, skin and thoracic and cervical spinal cord.

## Results and discussion

### Overall virome analysis

**Fig. 2.**
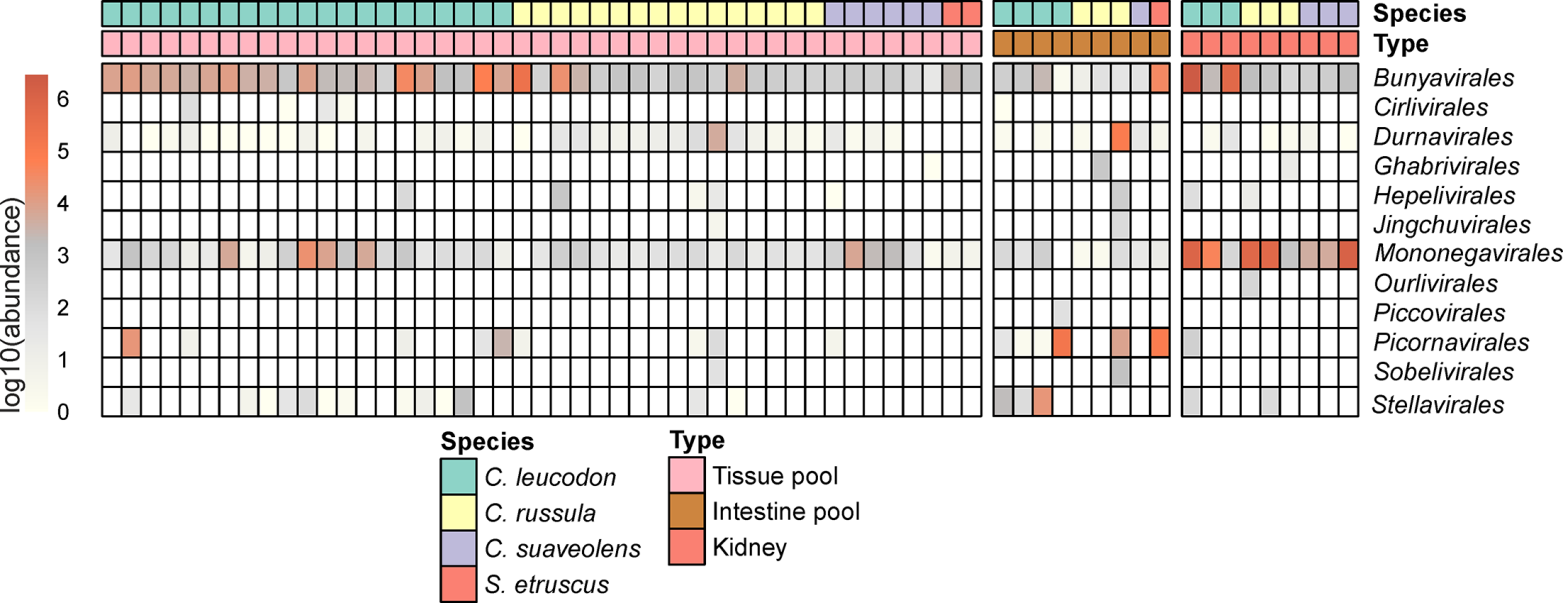
Viral diversity in different samples from white-toothed shrews. The heatmap shows the relative abundance of viral sequences sorted taxonomically by viral order. Note the abundance of sequence reads of the orders *Bunyavirales*, *Mononegavirales*, *Hepelivirales*, *Picornavirales* and *Stellavirales. Bunyavirales* and *Mononegavirales* were the most abundant orders in the tissue pools, while reads of *Picornavirales* and *Stellavirales* were predominantly detected in the intestine pools. Based on the observed tissue distribution, kidney tissue from selected individuals was additionally sequenced and included in the analysis.

Metagenomic analysis of tissue and intestine pools from a selected subpopulation of 45 white-toothed shrews collected across Europe (Fig. S1 and Table S1) revealed the presence of a wide range of RNA viruses belonging to the orders *Bunyavirales*, *Mononegavirales*, *Hepelivirales*, *Picornavirales* and *Stellavirales* (Fig. 2). Sequence reads of the orders *Bunyavirales* and *Mononegavirales* were the most abundant ones in the individual-based organ pools, while *Picornavirales* and *Stellavirales* were predominantly detected in the species-based intestine pools. Tissue pools provide the benefit of reduced sampling and sequencing bias due to non-homogeneous virus distribution in the different tissues. After initial sequencing, the libraries were further enriched for a broad range of epizootic and zoonotic viruses using capture enrichment with the VirBaits 2.0 myBait panel (Wylezich *et al*., unpublished).

Subsequent analysis focused on virus genera with public health implications [1,7]. In particular, we identified the whole genome sequences of novel orthoparamyxo-, orthonairo- and orthohepeviruses, as well as several complete genome sequences of the zoonotic BoDV-1 and ERVEV [14,18]. Virus-specific RT-qPCRs were designed in order to determine viral RNA tissue distribution. Based on the observed tissue distribution, kidney tissue from selected individuals was additionally sequenced and included in the analysis. The following sections summarize the results for each virus family.

### Detection and analysis of novel paramyxoviruses

Within the family *Paramyxoviridae* (order *Mononegavirales*), there are currently 4 subfamilies with 14 genera established [44]. The subfamily *Orthoparamyxovirinae* comprises several viruses with high impact on human and animal health, such as members of the genera *Morbillivirus* (measles virus and rinderpest virus, the first successfully eradicated epizootic disease) and *Henipavirus* (NiV, HeV), with reoccurring outbreaks of NiV, demonstrating dramatic case fatality rates of 40–70%, including possible human-to-human transmission [1,28, 45].

**Fig. 3.**
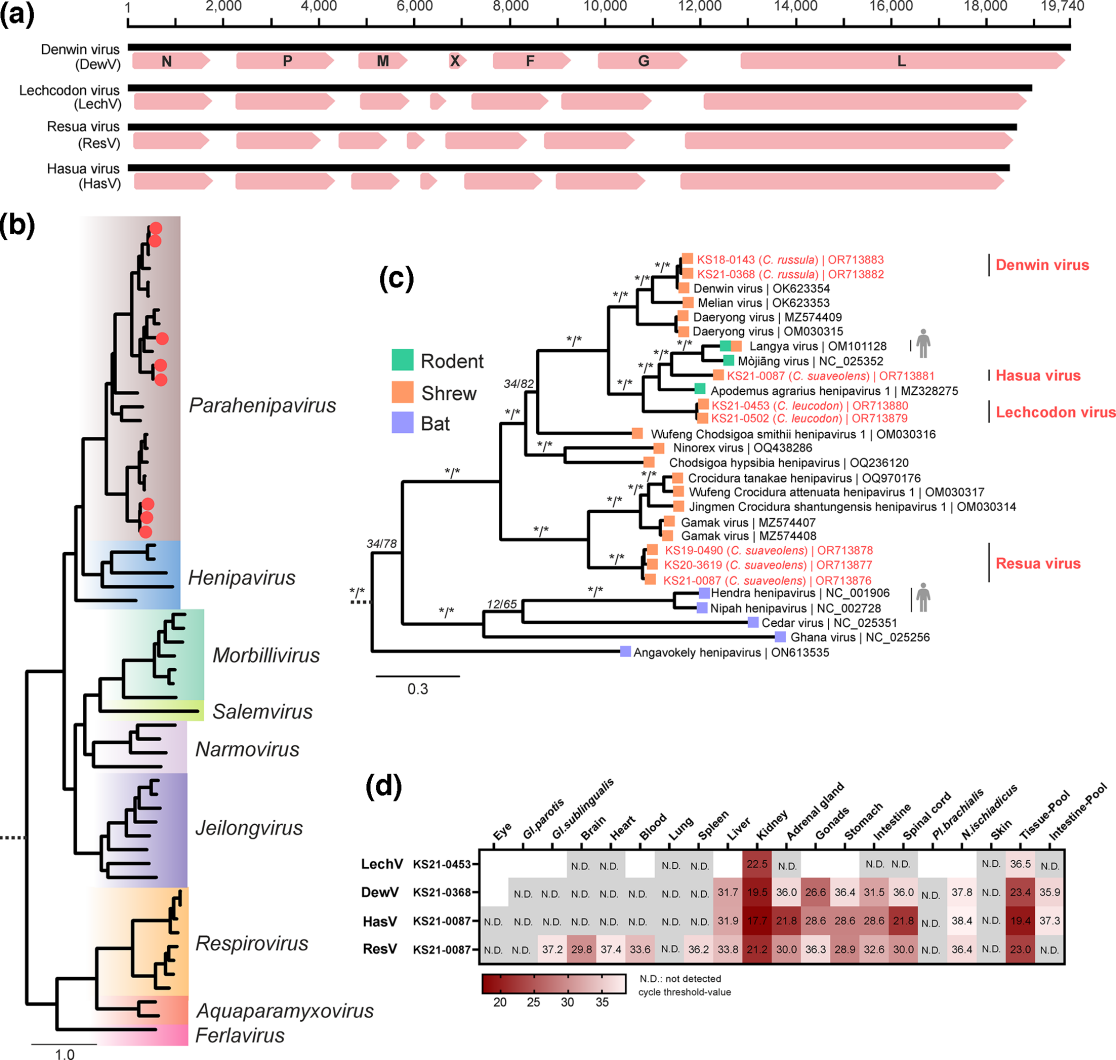
Detection and analysis of shrew-associated paramyxoviruses. (**a**) The genome structure of the novel paramyxoviruses was similar to that of Denwin virus, with the presence of the hypothetical open reading frame ‘X’, specific to other shrew-derived paramyxoviruses. (**b**) For phylogenetic analysis, we selected 54 representative genomes from the *Orthoparamyxovirinae* subfamily, using *Metaparamyxovirinae* as an outgroup. The amino acid sequences of the large protein (L, including RNA-directed RNA polymerase, capping and cap methylation activities) were aligned and used for phylogeny (IQ-TREE2; version 2.2.2.6). (**c**) Phylogenetic analysis of the genera *Henipavirus* and *Parahenipavirus*. Novel whole genomes are highlighted in red, and host-association is indicated by tip colour. Viruses with described zoonotic potential are highlighted with a human silhouette. Statistical support is shown for main branches using the format [SH-aLRT (%)/ultrafast bootstrap (%)]. Asterisks indicate statistical support ≥80 % and ≥ 95 % for ultrafast bootstrap and SH-aLRT, respectively. (**d**) Tissue distribution of paramyxovirus RNA using RT-qPCR specific for the L gene region. Results are given in cycle threshold values. No tissue was available for blank fields.

Within the tissue pools, we identified genomes (Fig. 3a) of diverse orthoparamyxoviruses that phylogenetically clustered within the newly established genus *Parahenipavirus* (Fig. 3b), extending this shrew-dominated group (Fig. 3b, c) [26,46]. The novel Hasua virus (HasV) was identified in *C. suaveolens* (KS21-0087) from north-eastern Germany and was phylogenetically closely related to LayV and Mòjiāng virus (MojV). When comparing the aa sequence of the large protein (L) of HasV to LayV and MojV, the identity ranged between 81.0 and 81.5 % (Fig. S2).

Interestingly, we found sequences of another novel orthoparamyxovirus, tentatively named Resua virus (ResV), in the same specimen (KS21-0087), suggesting co-infection. ResV was furthermore identified in two additional *C. suaveolens* from Germany (KS19-0490 and KS20-3619). ResV phylogenetically clustered with a distant group of exclusively shrew-derived paramyxoviruses, such as GamV. Lechcodon virus (LechV) was detected in two *C. leucodon* from southern Germany (KS21-0502, KS21-0453) and grouped basal to HasV and LayV (Figs 3c and S2).

Finally, sequences of the previously described DewV were detected in two *C. russula* (KS18-0143, KS21-0368), demonstrating its presence in Germany. In total, 9 out of 16 *C*. *russula* (56%, 95% confidence interval (CI): 33–77) were positive for DewV by RT-qPCR, indicating a high prevalence and wide geographical distribution of this virus (Table S1).

Virus-specific RT-qPCR confirmed the presence of these viruses, and viral RNA tropism was assessed, with high levels of viral RNA observed, particularly in kidney tissue. Although further assessment is needed, potential excretion and transmission via urine should be considered when establishing preventive measures (Fig. 3d). Efficient transmission via urine was demonstrated for HeV and NiV, even allowing direct bat-to-human transmission for NiV through the consumption of urine-contaminated food [28]. Otherwise, transmission of HeV and NiV from their fruit bat reservoir to humans requires an intermediate host, either horses or pigs, respectively [28].

The zoonotic potential of these novel paramyxoviruses cannot be addressed in this study, as further *in vitro* and *in vivo* downstream characterizations are required [6]. However, their phylogenetic proximity to known zoonotic agents (e.g. LayV) and to viruses that, at least experimentally, have the ability to infect human cells (e.g. GamV) [23,26] clearly warrants such work. These newly identified paramyxoviruses confirm the presence of a phylogenetically related group of shrew-derived viruses that form a sister clade to the bat-borne henipaviruses and support the increasing number of globally distributed paramyxoviruses [23,25,47].

### Detection and analysis of novel nairoviruses

The genus *Orthonairovirus* belongs to the family *Nairoviridae* of the order *Bunyavirales*. Orthonairoviruses are arthropod-borne, globally distributed viruses with a wide range of hosts, including mammals, birds and reptiles. In some cases, they can cause severe or fatal disease in livestock and wildlife, with substantial economic and ecological implications [3,20]. The reservoir species for many of these viruses have not been successfully identified yet, and small mammals have been considered putative reservoirs or amplification hosts [21]. Orthonairoviruses possess a negative-sense trisegmented RNA genome with a small (S) segment encoding the nucleoprotein, the medium segment encoding the glycoprotein precursor and the large segment encoding the large protein (L) which mediates viral replication and transcription [48].

In the sampled shrew tissue pools, orthonairovirus-related sequences were highly abundant and detected in 10 out of 45 tissue pools (22.2%) across *C. leucodon, C. russula* and *S. etruscus*, but were absent in the analysed *C. suaveolens*.

**Fig. 4.**
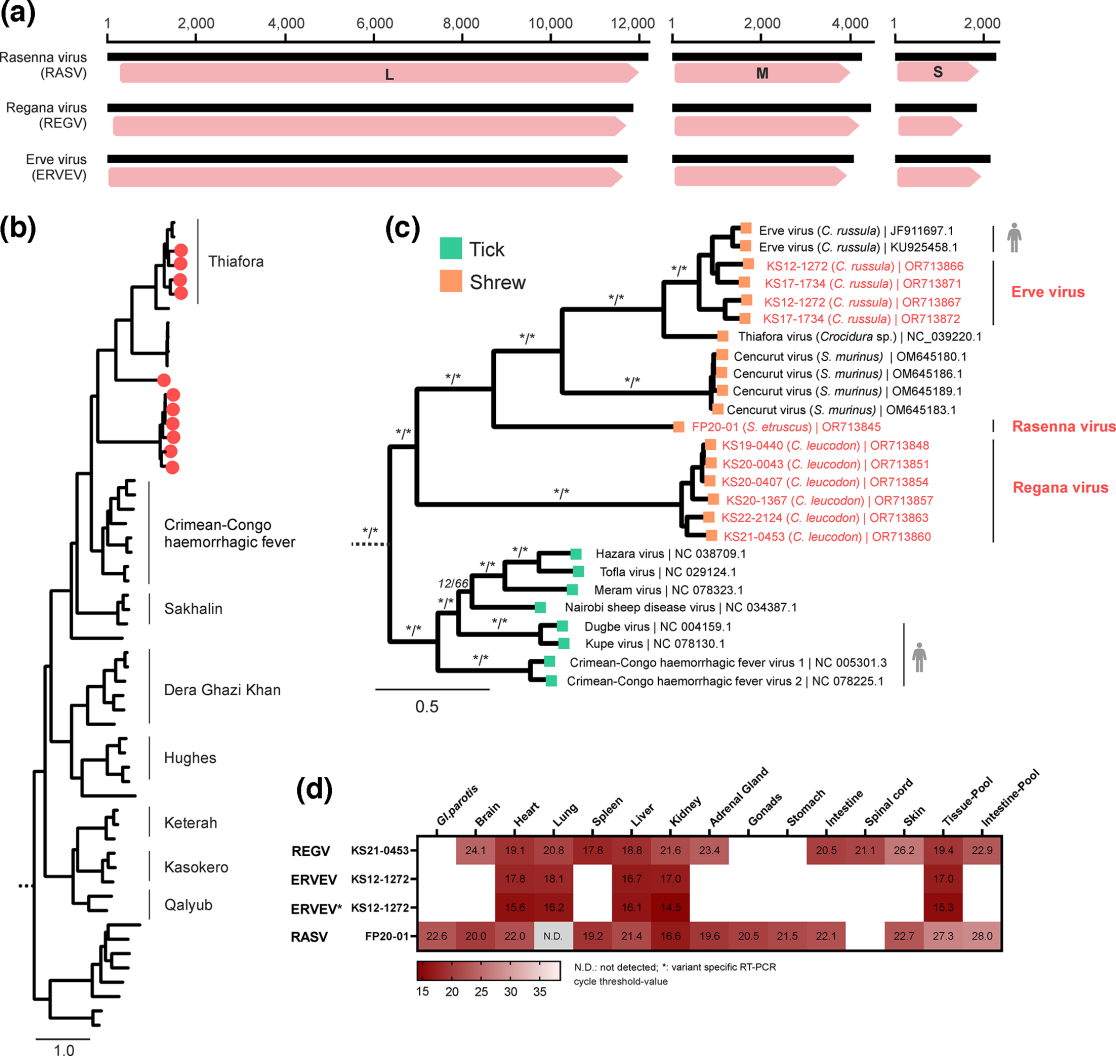
Detection and analysis of shrew-associated orthonairoviruses. (**a**) The segmented genome of the novel orthonairoviruses matched the size of the genomes of other members of the family *Nairoviridae*: the small (S) segment encoding for the nucleoprotein and the non-structural NSs, the medium (M) segment encoding for the glycoprotein precursor and the large (L) segment encoding for the RNA-directed RNA polymerase. (**b**) For the phylogeny of orthonairoviruses, we selected 46 representative genomes of the genus *Orthonairovirus* and one genome of the genus *Shaspivirus* as outgroup. The amino acid sequences of the RNA-directed RNA polymerase were aligned and used for phylogeny (IQ-TREE2; version 2.2.2.6). Novel genomes are indicated as red dots. (**c**) Detailed view of the phylogenetic relationships within the Crimean-Congo haemorrhagic fever and Thiafora virus genogroups. Newly generated whole genomes of Erve virus, Rasenna virus and Regana virus are shown in red. Host association is indicated by colour. Viruses with described zoonotic potential are highlighted with a human silhouette. Statistical support is shown for main branches using the format [SH-aLRT (%)/ultrafast bootstrap (%)]. Asterisks indicate statistical support ≥80% and ≥ 95% for ultrafast bootstrap and SH-aLRT, respectively. (**d**) Viral RNA tissue distribution as determined by virus-specific RT-qPCRs. KS12-1272 was tested with two different primers and probe sets to differentiate between the two strains of Erve virus. Results are given in cycle-threshold values. No tissue was available for blank fields.

Several phylogenetically distinct complete sequences of viral genome segments could be deduced (Figs2  4a) and phylogenetically grouped within the Thiafora virus genogroup, which includes the shrew-borne ERVEV, TFAV and CENV (Fig. 4b).

In detail, six whole genomes of the novel Regana virus (REGV) were identified exclusively in *C. leucodon* (KS19-0440, KS20-0043, KS20-0407, KS20-1367, KS21-0453 and KS22-2124) from all over Germany and the Czech Republic. REGV forms a monophyletic cluster basal to the known viruses within the Thiafora virus genogroup (Fig. 4c). Furthermore, several segments of ERVEV were identified in two tissue pools from *C. russula* (KS12-1272 and KS17-1734), and the novel Rasenna virus (RASV) was identified in captive *S. etruscus* (FP20-1), with its sequences clustering between REGV and CENV (Fig. 4c).

The aa sequence similarity of the L-protein between the identified REGV and ERVEV strains ranged between 84.4–99.3% and 79.6–90.2% (Fig. S3), respectively, which is below the ICTV species delimitation threshold of <93% for members of different species in the genus *Orthonairovirus* [48]. However, because of their geographic proximity and common host species, we tentatively considered all of these viruses to be variants of REGV or ERVEV, rather than proposing them as unique viruses.

The presence of multiple ERVEV variants within a single animal was subsequently confirmed using genome-specific RT-qPCR assays (Fig. 4d). This finding indicated the potential for reassortment, which is a process that can result in high genetic variability facilitating host adaptation or host species switches, commonly seen in segmented viruses such as those of the order *Bunyavirales* [21,49].

Virus-specific RT-qPCR analyses confirmed the presence of the new virus genomes in the tissue pools and in individual tissues, suggesting a broad tissue distribution with high viral RNA loads, especially in well-perfused organs. Liver tissue yielded the lowest cycle threshold values in all individuals (Fig. 4d). These findings are in accordance with a viraemic status of orthonairovirus infection and may indicate its circulation in the bloodstream. The putative role of ticks in the transmission of the orthonairoviruses detected remains a question for further study. However, the presence of RASV in captive *S. etruscus* from a well-established breeding colony suggests arthropod-independent transmission, as these animals were kept in a controlled ectoparasite-free environment [50]. Vertical and efficient direct shrew-to-shrew transmission via scratching and biting during territorial fights may be assumed for the stable viral persistence in the colony and the wild [8]. Isolation of these viruses may improve the knowledge of virus transmission, but attempts to isolate RASV in Vero and BHK-21 cells have not been successful.

The zoonotic potential of these novel viruses is currently unknown; however, ERVEV has been associated with reports of thunderclap headaches in humans [18,51]. The presence of genetically diverse ERVEV and the identification of new shrew-borne orthonairoviruses (REGV in *C. leucodon* and RASV in *S. etruscus*) demonstrate the high diversity of orthonairoviruses in white-toothed shrews.

### Detection and analysis of a novel hepevirus

Orthohepeviruses infect a wide range of species, including humans, pigs, rabbits, rodents, bats and birds. Generally, they are highly host-specific with the exception of zoonotic viruses of the genus *Paslahepevirus* [52]. Human hepatitis E virus (HEV; species *Paslahepevirus balayani*) can be transmitted faecal-orally through contaminated water and the consumption of undercooked meat products. It is a major cause of self-limiting acute hepatitis in humans, but can also induce severe chronic hepatitis in immunocompromised patients [53].

**Fig. 5.**
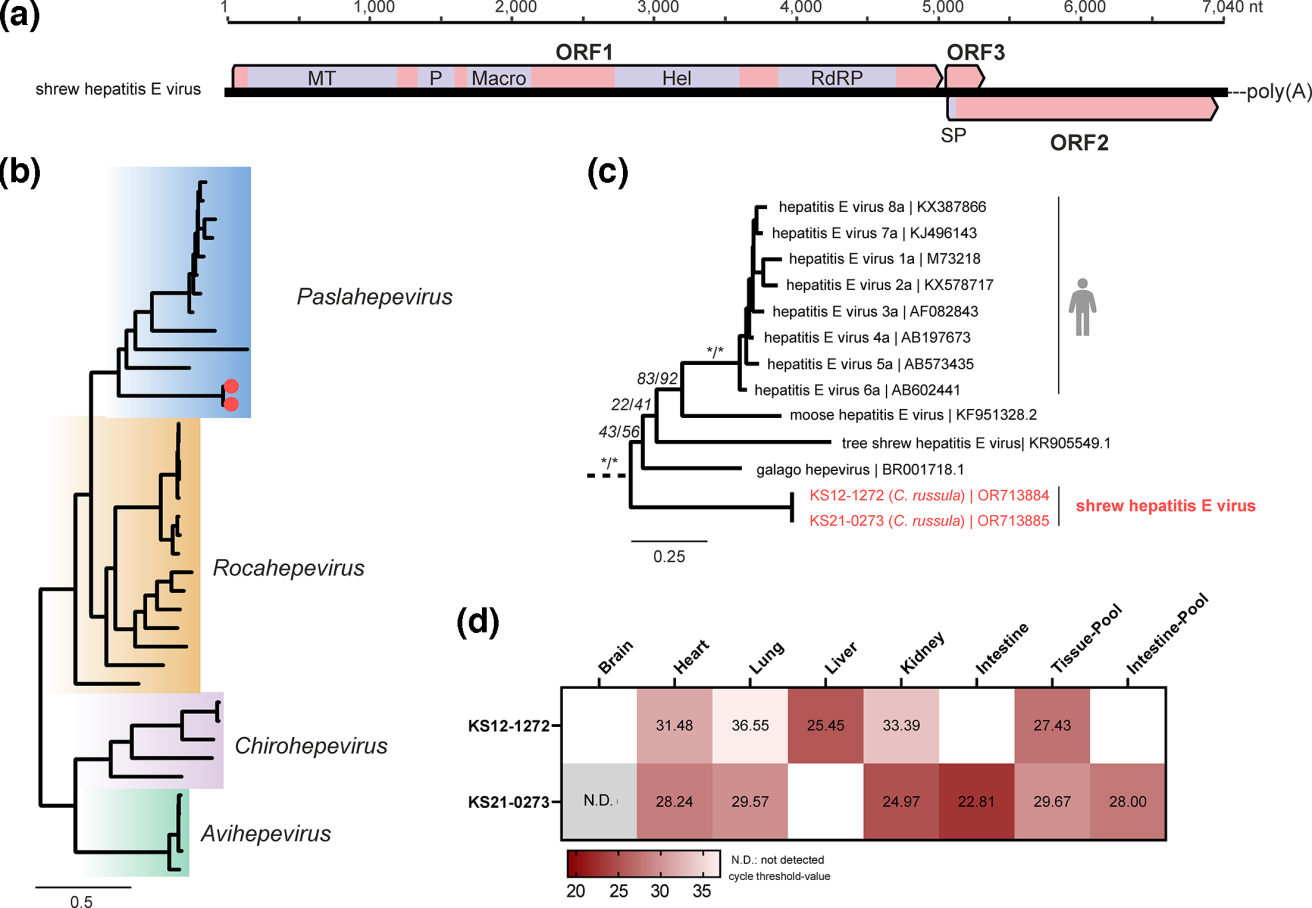
Detection and analysis of shrew-associated hepevirus. (**a**) Genome structure of the novel shrew hepatitis E virus. (**b**) For the phylogenetic analysis of the novel hepevirus, 36 representative genomes of the subfamily *Orthohepevirinae* and five genomes of fish hepeviruses (subfamily *Parahepevirinae*) were selected as references. The first 450 amino acids of ORF1-encoded non-structural polyprotein were aligned, and a phylogenetic tree was calculated (IQ-TREE2; version 2.2.2.6). Novel genomes are indicated as red dots. (**c**) Detailed view on the phylogenetic relations within the genus *Paslahepevirus*. Viruses with described zoonotic potential are highlighted with a human silhouette. The novel hepevirus sequences are indicated in red. Statistical support is shown for main branches using the format [SH-aLRT (%)/ultrafast bootstrap (%)]. Asterisks indicate statistical support ≥80% and ≥ 95% for ultrafast bootstrap and SH-aLRT, respectively. (**d**) Viral RNA tissue distribution of the novel shrew hepatitis E virus in two *C. russula* (KS12-1272, KS21-0273), as detected by virus-specific RT-qPCR. Results are given in cycle threshold values. No tissue was available for blank fields.

Two closely related whole genomes of a novel hepevirus of the subfamily *Orthohepevirinae* were identified in two specimens of *C. russula* (KS12-1272, KS21-0273) captured in western and eastern Germany (Table S1). They show a genome organization most similar to viruses of the genus *Paslahepevirus*, with an overlapping region of ORF2/ORF3, and the absence of ORF4, an ORF identified in rat hepatits E virus (Fig. 5a) [54]. This similarity in genome organization is reflected in the phylogenetic position of shrewHEV, which clusters with strains of the genus *Paslahepevirus* well separated from strains of the genus *Rocahepevirus* (Fig. 5b). The aa sequence of the first 450 aa of the ORF1-encoded non-structural polyprotein of the novel shrewHEV showed an identity between 55.7 and 61.9% in comparison to members of the genus *Paslahepevirus* and between 53.1 and 58.4% to members of the genus *Rocahepevirus* (Fig. S4). The highest viral RNA loads were detected in the liver tissue of KS12-1272 and in the kidney and intestinal tissue of KS21-0273, suggesting faecal-oral transmission (Fig. 5c).

### Detection and analysis of Borna disease virus 1

BoDV-1 belongs to the genus *Orthobornavirus* (family *Bornaviridae*). It causes sporadic but fatal encephalitis in domestic animals, primarily horses, sheep and New World camelids, and was only confirmed as zoonotic in 2018 [14,42]. The transmission from its reservoir to dead-end hosts, its presence in the reservoir population and the appearance of its endemic area are still poorly understood.

In this study, we generated seven new BoDV-1 complete genome sequences from four *C. leucodon* and, for the first time, from two *C. suaveolens* and one *C. russula* (Fig. S5). In accordance with their geographic origin, these new BoDV-1 sequences fall within the established phylogeographic clusters [14,55]. The presence of BoDV-1 RNA in the tissue pools was confirmed by specific RT-qPCRs.

### Co-infection of different viruses

Several shrews in the study demonstrated co-infections with multiple viruses. For example, *C. russula* KS12-1272 was found to carry three viruses: shrewHEV (complete genome identified), ERVE (two different complete L segments detected) and DewV (detected by RT-qPCR). Similarly, *C. suaveolens* KS21-0087 showed a triple infection containing two distinct paramyxoviruses (HasV and ResV) and BoDV-1. In addition to the complete genome of shrewHEV, DewV was also identified by RT-qPCR in *C. russula* KS21-0273. *Crocidura russula* KS21-0368 tested positive for both DewV and BoDV-1, while *C. suaveolens* KS20-3619 tested positive for BoDV-1 and ResV. The complete genomes of LechV and REGV were present in *C. leucodon* KS-0453. BoDV-1 and REGV were identified in both *C. leucodon* specimens (KS20-1367 and KS21-0392), as detailed in Fig. S6.

### Influence on virus sequencing efficiency using bait-captured libraries

The influence on virus-to-background ratio by application of VirBaits 2.0 capture enrichment was virus-specific. For BoDV-1, the efficiency was highly increased, while for the newly discovered viruses, the virus-to-background ratio was only minimally improved, if at all (Fig. S7). This can be explained as the used capturing probes were tailored to the genetic sequences of known viruses (e.g. CCHFV, HeV, NiV, HEV and BoDV-1), enhancing their binding efficiency and capture performance for those targets. In the case of BoDV-1, the probes are likely to have high affinity and specificity, resulting in a significant increase in the virus-to-background ratio. This means that the probes effectively capture the viral sequences, reducing the amount of non-target, or background, sequences in the library, thereby improving sequencing efficiency. On the other hand, for newly discovered viruses that are highly divergent from the references on the nucleotide level, the probes used in the bait set might be less specific, and efficiency is decreased. This is a common challenge in capture enrichment technologies, where the effectiveness of the probes depends heavily on their sequence complementarity to the target viruses [31].

## Conclusion

Investigations of species-rich and phylogenetically ancient wildlife taxa such as shrews improve our understanding of global virus distribution. Revisions to existing taxonomy and the continued discovery of new shrew species, as well as the expansion of the range of some shrew species, demonstrate the high complexity of this group of animals. There is limited information available on the basic parameters of shrew’s biology, such as population structure and dynamics. However, shrews may share similar properties with other so-called viral hyperreservoirs, such as bats and rodents. Their high metabolism, torpor, fast life cycle and unknown immunological responses to viral infection may enable them to sustain and spread viral infections without developing any disease.

Viruses detected in *C. russula*, which has a North African origin, are genetically similar to other viruses detected in African shrews (ERVEV and TFAV for nairoviruses, and DewV and MeliV for paramyxoviruses), whereas *C. suaveolens*, which is widely distributed across Eurasia, presented viruses with close relatives detected in Asian shrews (HasV and LayV). This suggests a certain degree of co-evolution between the shrew species and their carried viruses.

In the context of increased pandemic preparedness, these viruses and their reservoirs need to be studied in more detail to assess their pathological relevance, mode of transmission and potential as surrogates for vaccine development. Although the elusive behaviour of these synanthropic shrews makes it difficult to grasp the human–shrew interface, it does exist, as evidenced by human BoDV-1 infections. In any case, the findings indicate the need for biosafety considerations when handling wild animals of these species.

Our results demonstrate the great diversity of viruses harboured in wildlife, not only in biodiversity hotspots but also in the human-dominated landscapes of Europe. In a holistic One Health approach, future studies should evaluate the potential influence of anthropogenic land use, biodiversity and climate change on the range of these neglected reservoir species and their potential as reservoirs while acknowledging the conservation of white-toothed shrews.

## supplementary material

10.1099/mgen.0.001275 Supplementary Material 1.

10.1099/mgen.0.001275 Supplementary Material 2.

## Abbreviations

BoDV-1: Borna disease virus 1
CCHFV: Crimean-Congo haemorrhagic fever virus
CENV: Cencurut virus
DewV: Denwin virus
ERVEV: Erve virus
GamV: Gamak virus
HasV: Hasua virus
HeV: Hendra virus
ICTV: International Committee on Taxonomy of Viruses
LayV: Langya virus
LechV: Lechcodon virus
MeliV: Melian virus
MojV: Mòjiāng virus
NiV: Nipah virus
ORF: open reading frame
RASV: Rasenna virus
REGV: Regana virus
ResV: Resua virus
RT-qPCR: quantitative reverse transcription PCR
TFAV: Thiafora virus
