# Supplementary material for: Detection of novel orthoparamyxoviruses, orthonairoviruses and an orthohepevirus in European white-toothed shrews
Source: Microb Genom. 2024 Aug 1;10(8):001275. doi: 10.1099/mgen.0.001275 (PMC11293873; doi:10.1099/mgen.0.001275)
Supplement: Supplementary Material 1. [file mgen-10-01275-s001.pdf]

Supplementary Material

for

Detection of novel orthoparamyxoviruses, orthonairoviruses and an orthohepevirus in European white-toothed shrews

Haring *et al.* 2024

Supplementary Figures

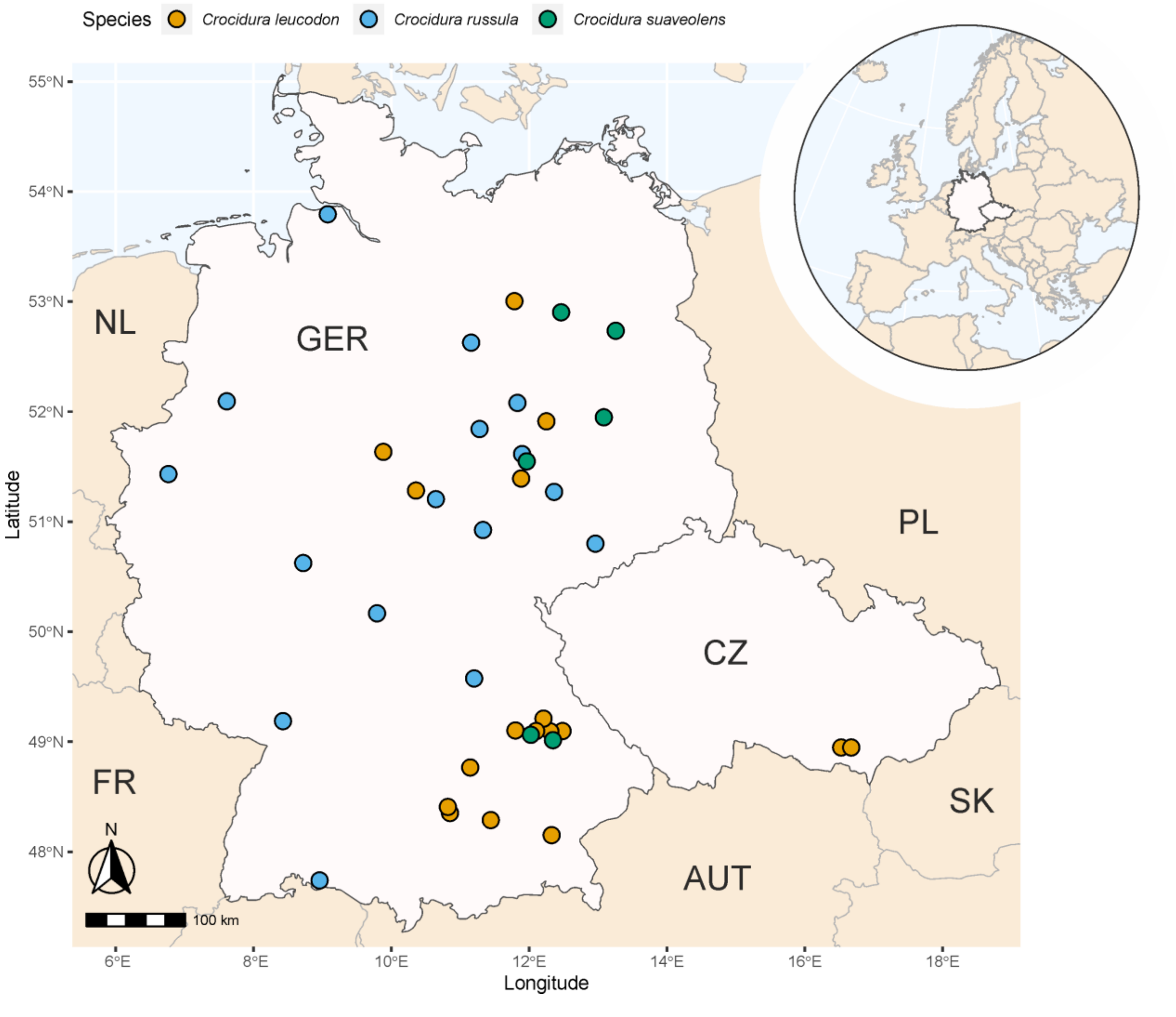

Supplementary Figure S1: Geographical origin of studied white-toothed shrews (n=45) from Germany and the Czech Republic. This study included 19 bicolored white-toothed shrews (*Crocidura leucodon*), 16 greater white-toothed shrews (*Crocidura russula*), and six lesser white-toothed shrews (*Crocidura suaveolens*) from Germany (GER) and two *C. leucodon* collected in the Czech Republic (CZ). Two additional Etruscan shrews (*Suncus etruscus*) originated from a colony in Germany and are therefore not shown. NL: the Netherlands; FR: France; AUT: Austria; SK: Slovakia; PL: Poland.

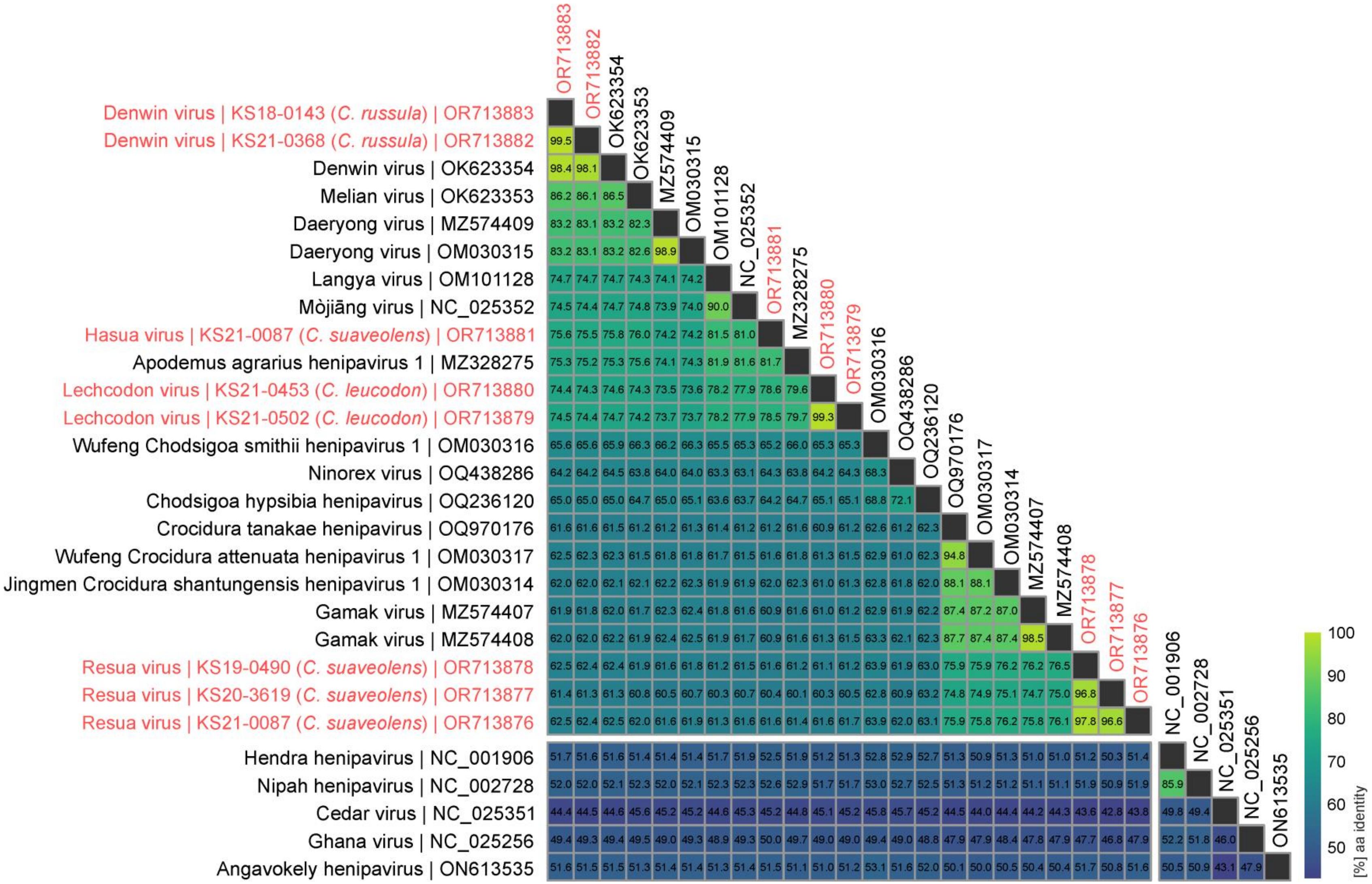

Supplementary Figure S2: Heatmaps show L-protein amino acid (aa) sequence similarity between henipaviruses and parahenipaviruses. Novel viruses identified in this study are indicated in red.

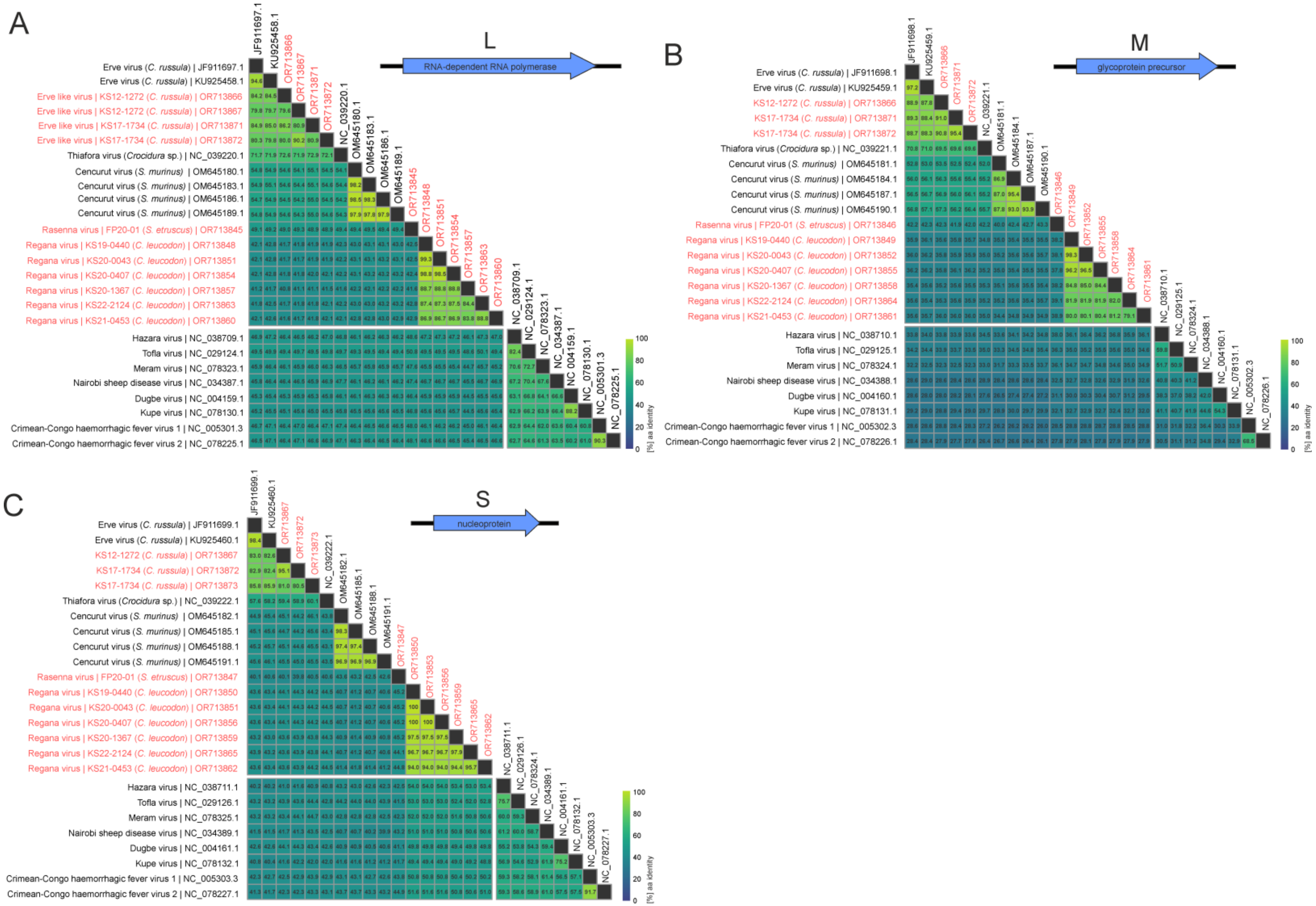

Supplementary Figure S3: The heatmaps demonstrate amino acid (aa) sequence similarity of the proteins encoded by L-segment (**A**), M-segment (**B**) and S-segment (**C**) of orthonairoviruses. Novel viruses identified in this study are indicated in red.

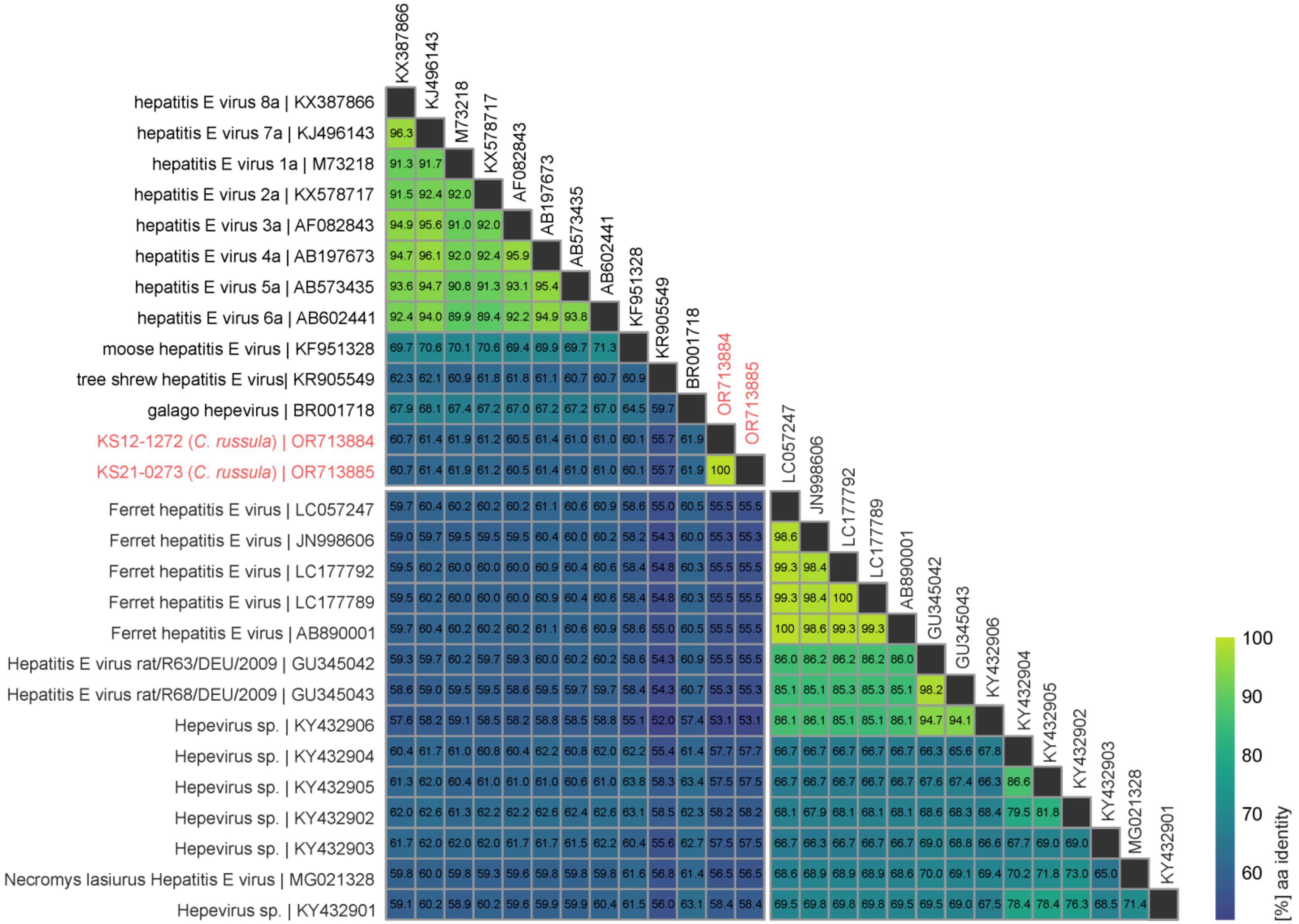

Supplementary Figure S4: Heatmap representing the amino acid (aa) sequence similarity of the first 450 aa of ORF1-encoded non-structural polyprotein for members of the genera *Paslahepevirus* and *Rocahepevirus*. The novel virus identified in this study is indicated in red.

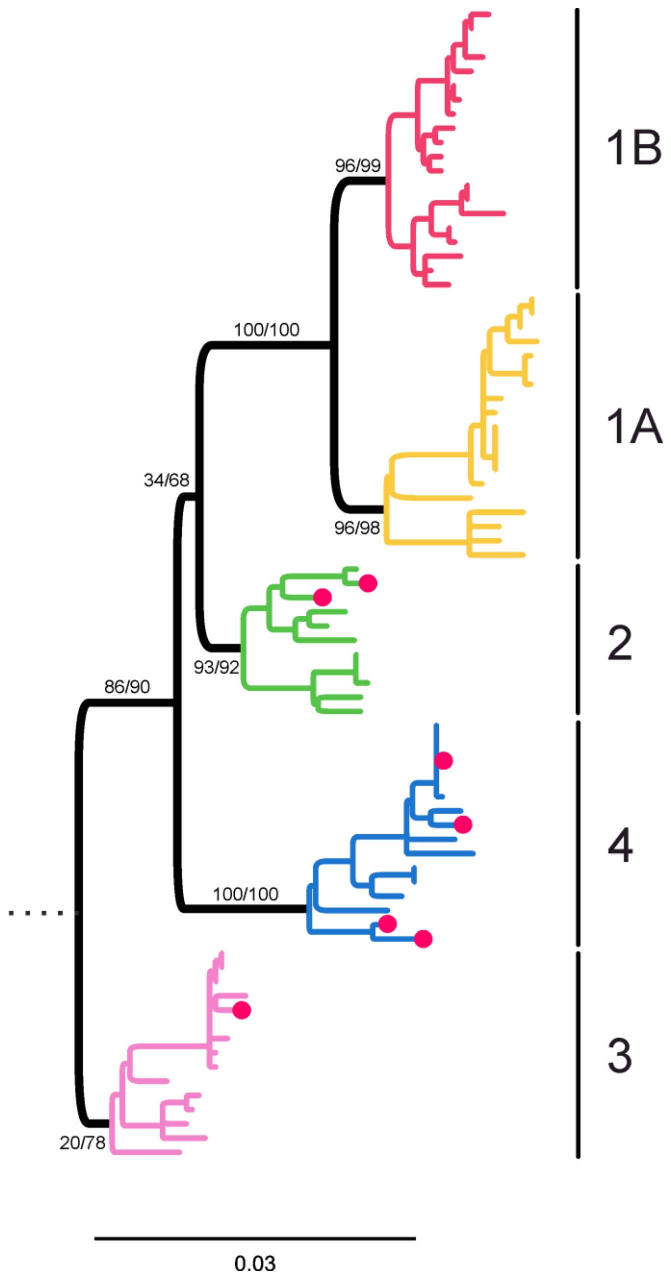

Supplementary Figure S5: Phylogenetic relationships of the identified Borna disease virus 1 (BoDV-1) strains. For phylogenetic analysis, we selected 74 representative sequences of BoDV-1 together with the strains identified in this study (red dots). BoDV-2 was used as an outgroup but is not shown. Only the NX/P-region was selected and aligned using MUSCLE. Maximum likelihood phylogeny was inferred using IQ-TREE2 (version 2.2.2.6) with an automated model selection and 100,000 ultra-fast bootstrap and SH-aLRT replicates each. Only bootstrap values at major branches are shown. Established BoDV-1 clusters are highlighted in different colours.

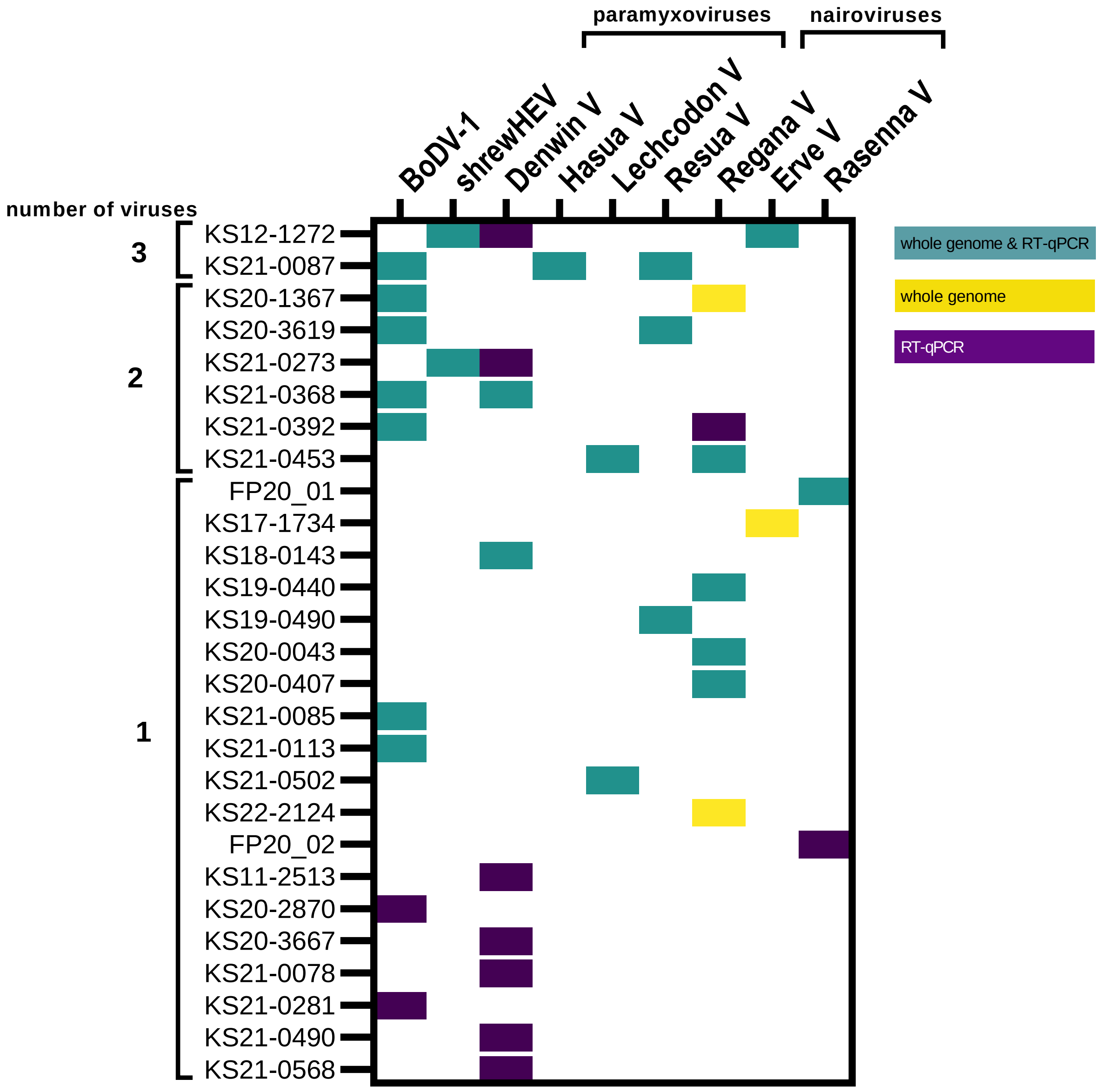

Supplementary Figure S6: Co-detection of up to three viruses from different families in white-toothed shrews. Samples are sorted by the number of identified pathogens. Samples from which a viral whole genome sequence could be derived and corresponding positive RT-qPCR results are shown in turquoise, while virus identification by RT-qPCR alone is shown in purple. Three samples shown in yellow had whole genome sequences only.

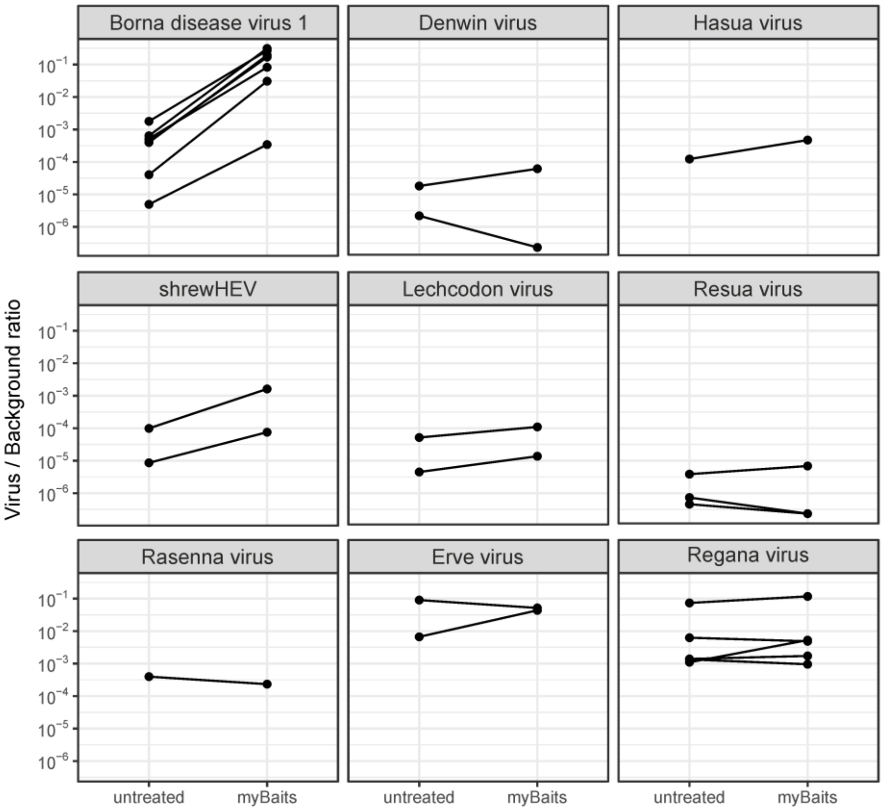

Supplementary Figure S7: The influence of the application of VirBaits 2.0 capture enrichment on Virus-to-Background ratio for high throughput-sequencing libraries. The VirBaits 2.0 bait set comprises 134,710 RNA baits specific to numerous epizootic and zoonotic viruses.

Supplementary Tables

Supplementary Table S1: Information on the investigated white-toothed shrews, pool composition, sequencing information and detected viruses. See file "Supplementary Table S1.xlsx".

Supplementary Table S2: Detailed information on the detected viruses in the white-toothed shrew samples

Taxonomic classification	Virus name	Tentatively named after	Detected in	Whole genomes generated	Additional RT-qPCR positive individuals
Paramyxoviridae: Parahenipavirus	Hasua virus (HasV)	river "**Ha**vel" and the species abbreviation "*C.* ***sua****veolens*"	*C. suaveolens*	KS21-0087	-
	Resua virus (ResV)	river "**Re**gen" and the species abbreviation "*C.* ***sua****veolens*"	*C. suaveolens*	KS19-0490, KS21-0087, KS20-3619	-
	Lechcodon virus (LechV)	river "**Lech**" and the species abbreviation "*C. leu****codon***"'	*C. leucodon*	KS21-0453, KS21-0502	-
	Denwin virus (DewV)		*C. russula*	KS18-0143, KS21-0368	KS11-2513, KS12-1272, KS20-3667, KS21-0078, KS21-0273, KS21-0490, KS21-0568
Nairoviridae: Orthonairovirus	Rasenna virus (RASV)	named after the Etruscan civilization's own designation "**Rasenna"**	*S. etruscus*	FP20-01	FP20-02
	Erve virus (ERVEV)		*C. russula*	KS12-1272, KS17-1734	-
	Regana virus (REGV)	**"Regana"** is the Germanic word for "Regen", the nearby river in the Bavarian region where most of the positive shrews were caught	*C. leucodon*	KS19-0440, KS20-0043, KS20-0407, KS20-1367, KS22-2124, KS21-0453	KS21-0392
Hepeviridae: Paslahepevirus	shrew hepatitis E virus (shrewHEV)	Phylogenetic relationship to hepatitis E virus	*C. russula*	KS12-1272, KS21-0273	-
Bornaviridae: Orthobornavirus	Borna disease virus 1 (BoDV-1)		*C. leucodon*	KS20-1367, KS21-0085, KS21-0113, KS21-0392	KS21-2870, KS21-0281
			C. russula	KS21-0368	
			C. suaveolens	KS21-0087, KS20-3619	

Supplementary Table S3: Primer and probes used for RT-qPCRs

Name*	Type	Sequence (5'→3')	Reference
ReganaV-1-F	Primer	TCTCCACCTGCCTACAGAGA	This study
ReganaV-1-R	Primer	TGCTGCTCTTTCTTTTCAGGA	This study
ReganaV-1-FAM	Probe	FAM-TGCAGCAGATACTGATGGATTTCCCA-BHQ-1	This study
ReganaV-4-F	Primer	CCAGAACCTAAACAGAGCATTC	This study
ReganaV-4-R	Primer	ACCAAATCCCACATCTGCTATA	This study
ReganaV-4-FAM	Probe	FAM-AATGAAGAACGCCCTTTACTTAGTGTGTG-BHQ-1	This study
ErveV-1-F	Primer	TAGAAGGTCAAGCTCATCGAAT	This study
ErveV-1-R	Primer	ACTCAAGGAAAATGCCAGAATC	This study
ErveV-1-FAM	Probe	FAM-ACCTCAAGTCTGATCAATAGATACACCACC-BHQ-1	This study
ErveV-2-F	Primer	AGAGGAGTTGGACAATAGGATG	This study
ErveV-2-R	Primer	CTTCAAAATGCCCATTCAACAC	This study
ErveV-2-FAM	Probe	FAM-CTGCTGACAAATTTTATAACTGAGGCGGTA-BHQ-1	This study
RasennaV-1-F	Primer	AACGCATCATGAATGGCCACA	This study
RasennaV-1-R	Primer	CAAACCCAGTGGTAAGCAGCA	This study
RasennaV-1-FAM	Probe	FAM-CCACCTTGGGGAGATGTGGATAAGCA-BHQ-1	This study
LechcodonV-F	Primer	TACATCCAACTGAACATGAACT	This study
LechcodonV-R	Primer	ATCATTAATCTCCCTTCAAGCA	This study
LechcodonV-FAM	Probe	FAM-GTGTCGTTGATTGGAATAGTCGAAAGATCT-BHQ-1	This study
DenwinV-F	Primer	CAGAAACAATAATCAGTACACATTTC	This study
DenwinV-R	Primer	CACCTTGATATAGATTTTAGTCCC	This study
DenwinV-FAM	Probe	FAM-TTCCAAAAGGATTTACTATGATGGGATGGT-BHQ-1	This study
HasuaV-F	Primer	TCAGATAAACATGAACCTTGTC	This study
HasuaV-R	Primer	TCACAATACATGAGAACAAGTT	This study
HasuaV-FAM	Probe	FAM-GGAACAAGAAACAAATTTATCATTTAACACCA-BHQ-1	This study
ResuaV-F	Primer	TCAATCAAAATCTTTGCACCAT	This study
ResuaV-R	Primer	CCTTCAACAACATCACAATACA	This study
ResuaV-FAM	Probe	FAM-AGAGATTTACCATTTAACTCCTGAACTTGT-BHQ-1	This study
shrewHEV-F	Primer	CAGACGCGGTGGTTCAAAC	This study
shrewHEV-R	Primer	GTGGAACCAAGGGCAGCT	This study
shrewHEV-FAM	Probe	FAM-CCAGCCAGAGTCATTTCCACTAACAACCC-BHQ-1	This study
BoDV-1-1288-F	Primer	TAGTYAGGAGGCTCAATGGCA	(1)
BoDV-1-1449-R	Primer	GTCCYTCAGGAGCTGGTC	(1)
BoDV-1-1346-FAM	Probe	FAM-AAGAAGATCCCCAGACACTACGACG-BHQ1	(1)
ACT-1030-F	Primer	AGCGCAAGTACTCCGTGTG	(2)
ACT-1135-R	Primer	CGGACTCATCGTACTCCTGCTT	(2)
ACT-1081-HEX	Probe	HEX-TCGCTGTCCACCTTCCAGCAGATGT-BHQ1	(2)

*: F, forward; R, reverse

Supplementary References

1. Schlottau K, Forth L, Angstwurm K, Höper D, Zecher D, Liesche F et al. Fatal Encephalitic Borna Disease Virus 1 in Solid-Organ Transplant Recipients. The New England journal of medicine 2018; 379(14):1377–9.

2. Wernike K, Hoffmann B, Kalthoff D, König P, Beer M. Development and validation of a triplex real-time PCR assay for the rapid detection and differentiation of wild-type and glycoprotein E-deleted vaccine strains of Bovine herpesvirus type 1. J Virol Methods 2011; 174(1-2):77–84.
